# Effects of an Extensively Hydrolyzed Formula Supplemented with Two Human Milk Oligosaccharides on Growth, Tolerability, Safety and Infection Risk in Infants with Cow’s Milk Protein Allergy: A Randomized, Multi-Center Trial

**DOI:** 10.3390/nu14030530

**Published:** 2022-01-26

**Authors:** Yvan Vandenplas, Marta Żołnowska, Roberto Berni Canani, Siân Ludman, Zsuzsanna Tengelyi, Ana Moreno-Álvarez, Anne E. N. Goh, Maria Laura Gosoniu, Bridget-Anne Kirwan, Monika Tadi, Ralf G. Heine, CINNAMON Study Investigator Group

**Affiliations:** 1 UZ Brussel KidZ Health Castle, Vrije Universiteit Brussel, 1090 Brussels, Belgium; 2 Centrum Medyczne Plejady, 31-363 Kraków, Poland; marta.zolnowska@gmail.com; 3 Department of Translational Medical Science, University Federico II, 80131 Naples, Italy; berni@unina.it; 4 Royal Devon and Exeter NHS Foundation Trust, Exeter EX2 5DW, UK; sian.ludman@nhs.net; 5 Clinexpert Gyógycentrum, 1033 Budapest, Hungary; zstengelyi@gmail.com; 6 Department of Pediatrics, A Coruña University Hospital, 15006 A Coruña, Spain; Ana.Moreno.Alvarez@sergas.es; 7 KK Women’s and Children’s Hospital, Singapore 229899, Singapore; anne.goh.e.n@singhealth.com.sg; 8 Nestlé Research, 1800 Vevey, Switzerland; marialaura.gosoniu@rdls.nestle.com; 9 SOCAR Research, 1260 Nyon, Switzerland; bridget.kirwan@socar.ch; 10 Nestlé Health Science, 1800 Vevey, Switzerland; Monika.Tadi@rd.nestle.com (M.T.); Ralf.Heine@au.nestle.com (R.G.H.)

**Keywords:** 2′-fucosyllactose, lacto-N-neotetraose, food allergy, whey hydrolysate, respiratory infection, otitis media, gastroenteritis

## Abstract

This randomized clinical trial (Registration: NCT03085134) assessed if an extensively hydrolyzed formula (EHF) supplemented with two human milk oligosaccharides (HMO) and reduced protein content (2.20 g/100 kcal) supports normal growth in infants with cow’s milk protein allergy (CMPA). Secondary outcomes were gastrointestinal tolerability, safety, and effect on infections. Nonbreastfed infants aged 0–6 months with CMPA were enrolled. Body weight, length, and head circumference were measured monthly for 4 months (primary study endpoint), after 6 months, and at the age of 12 months. Of 200 infants screened, 194 (mean age 3.2 months) were randomized. At the 4-month follow-up, daily weight gain for the test formula was noninferior to the control formula; *p* < 0.005. There were no significant group differences in anthropometric parameters. Both formulas were safe and well tolerated. Infants in the HMO group had a statistically significant reduction in the frequency of upper respiratory tract infections and a lower incidence of ear infections at 12 months (per protocol analysis). The relative risk of lower respiratory tract and gastrointestinal infections was reduced by 30–40%, but this was not statistically significant due to sample size limitations. In summary, the HMO-supplemented formula supports normal growth in infants with CMPA and suggests a protective effect against respiratory and ear infections in the first year of life.

## 1. Introduction

Human milk oligosaccharides (HMO) are complex, nondigestible carbohydrates that make up the third biggest component of human milk solids [1]. HMO provide the host-specific substrate for the developing gut microbiome in early infancy, with targeted enhancement of bifidobacteria [2,3]. About 1–2% of HMO are absorbed and have systemic effects on immune function and even cognitive development [4,5,6,7]. Furthermore, HMO reduce infections in infancy by interfering with the mucosal attachment of respiratory or enteric pathogens. While the protection against infections in breastfed infants was previously attributed to the presence of maternal IgA antibodies in breast milk [8], there is now an increasing recognition of the anti-infective properties of HMO [9]. HMO reduce the risk of infection with a range of viral pathogens, including influenza virus, respiratory syncytial virus, coronaviruses, rotavirus, and norovirus [10,11,12,13]. There is also in-vitro evidence that HMO reduce the mucosal adhesion of bacterial pathogens, including *Streptococcus pneumoniae* and *Haemophilus influenzae* [14]. 

Maternal secretor status for the enzyme fucosyltransferase-2 (FUT2) is strongly associated with the composition of intestinal bifidobacteria in infants [15,16]. Breast milk of secretor mothers is high in 2′-fucosyllactose (2′-FL), which is the main substrate for several bifidobacterial species. By contrast, the gut microbiome of infants of nonsecretor mothers is low in bifidobacteria due to low levels of 2′-FL in breast milk, which may provide lower levels of biological protection against infections [17]. HMO levels in breast milk gradually decrease during lactation, however, in secretor mothers, significant levels of 2′-FL persist until at least 12 months after delivery [18]. Recently, 2′-FL and lacto-N-neotetraose (LNnT) became novel ingredients in infant formulas [19,20,21]. A randomized trial in healthy formula-fed infants showed that supplementation of standard formula with 2′-FL and LNnT was associated with a significant reduction in the incidence of lower respiratory tract infections and antibiotic use in the first year of life [22], in line with the HMO effects observed in breastfed infants [9]. No data on HMO effects in formula-fed infants with cow’s milk protein allergy (CMPA) are available to date.

Apart from the overall dietary energy provided to infants, the daily protein intake was identified as an important modifier of weight gain and obesity risk in infants and children [23,24,25]. Higher levels of dietary protein in the first six months of life are associated with increased levels of branched-chain amino acids and acylcarnitines, which stimulate beta-oxidation and fat storage [26]. Over the past two decades, many standard infant formulas were reduced in protein content in an attempt to reduce the risk of excess weight gain [24]. By contrast, most extensively hydrolyzed formulas (EHF) and amino acid-based formulas (AAF) provide a relatively high protein equivalent in the form of peptides or free amino acids to counteract potential malabsorption and provide adequate nutrients for catch-up growth [27]. It is unclear whether the higher protein content in EHF and AAF could be associated with increased weight gain in infants with CMPA.

In the present study, we aimed to assess whether the daily weight gain in infants with CMPA fed an EHF with a reduced protein content and supplemented with two HMO was noninferior to growth patterns observed for an existing EHF without HMO. In addition, we assessed the effects of the protein-reduced, HMO-supplemented formula on anthropometric parameters, tolerability, safety, infective morbidity, and medication use in the first year of life.

## 2. Materials and Methods

The present study was conducted in 41 clinical sites in Europe and 3 sites in Singapore between February 2017 and August 2018. Human ethics committee approval of the study protocol was obtained from each of the local Institutional review boards of the participating clinical centers. Written informed consent was obtained from parents or legal guardians of the participating infants. The study was prospectively registered (ClinicalTrials.gov; NCT03085134) and conducted in accordance with the principles and rules described in the Declaration of Helsinki and the international guideline on Good Clinical Practice (GCP). 

### 2.1. Study Design

This controlled, double-blind, randomized, multicenter, interventional clinical trial of two parallel formula-fed groups was designed to demonstrate noninferiority in weight gain per day in infants with CMPA being fed the test formula, compared to that of a reference control EHF with proven clinical efficacy [28,29]. Hypoallergenicity of the test formula was previously demonstrated [30]. 

### 2.2. Inclusion and Exclusion Criteria

Full-term infants aged 0–6 months with physician-diagnosed CMPA were enrolled. All infants were nonbreastfed at the time of enrollment. The diagnosis of CMPA required the presence of at least two of the following symptoms: persistent crying, frequent regurgitation, liquid stools, persistent constipation, atopic dermatitis, urticaria, or persistent respiratory symptoms. In the setting of either a positive cow’s milk-specific serum-IgE, positive skin prick test, or recent positive oral food challenge (OFC), only one of the previous symptoms needed to be present. Infants with a history of prior treatment with an EHF or AAF for more than 72 h were excluded. Further exclusion criteria included significant congenital illness or malformation that may affect growth, chronic malabsorption unrelated to CMPA, or a history of anaphylaxis to cow’s milk protein.

### 2.3. Study Formulas

The test formula was a 100% whey-based EHF supplemented with 2′-FL at a concentration of 1.0 g/L and LNnT at 0.5 g/L [30]. The control formula was a commercially available EHF without HMO, with documented adequate growth and hypoallergenicity in infants with CMPA (Althéra^®^, Nestlé Health Science, Vevey, Switzerland) [28,29]. The two study formulas were manufactured with a nonporcine enzyme blend. The protein/peptide content of the test formula was 2.20 g/100 kcal, compared to 2.47 g/100 kcal for the control formula. The macronutrient and micronutrient profiles of both formulas were otherwise almost identical. Both formulas contained highly purified lactose which provided 52% of total carbohydrates (3.8 g/100 mL formula). Taste and appearance of test and control formula were indistinguishable. Both formulas were provided in powder form and packaged in coded tins. Parents or caregivers were asked to follow general feeding advice regarding appropriate feeding volumes per day, as printed on the formula label or as provided by their health care professional.

### 2.4. Study Objectives and Endpoints

The primary objective of this study was to assess if infants with CMPA aged 0–6 months who were fed with the test formula achieved similar growth, compared to that of infants receiving the control formula. The primary study endpoint was noninferiority of the test formula for weight gain per day from enrollment to 4 months’ follow-up. Secondary objectives included the comparison of other growth parameters (weight, length, head circumference) with the WHO growth reference, as well as a comparison of symptom resolution between groups. The incidence of infections (respiratory, gastrointestinal, and other) and medication use (antibiotics, antipyretics) from enrollment to 12 months of age were compared between treatment groups.

### 2.5. Monitoring of Symptom Resolution

To assess the response to the study formula, investigators completed the cow’s milk-related symptom score (CoMiSS) at baseline and at each follow-up visit to V6 [31]. CoMiSS assesses 5 clinical domains (crying, regurgitation, stools, respiratory symptoms, and skin signs), with total scores ranging from 0 to 33. A score of 5 is considered normal in healthy European infants under 6 months of age [32]. While not a diagnostic tool, CoMiSS was found to be useful in the tracking of CMPA symptoms in response to a cow’s milk protein elimination diet [33,34,35].

### 2.6. Schedule of Study Visits

Infants were fed the study formulas from enrollment (Visit 0; V0) until 4 months (V4) postbaseline (primary study period). Growth parameters, clinical symptoms and CoMiSS score were documented at V0 and monitored monthly (from V1 to V4). Families were offered to continue the allocated study formula until 12 months of age, with additional follow-up visits at 6 months from enrollment (V5) and at 12 months of age (V6), prior to final discharge from the study. Unblinding of the treatment allocation occurred after all subjects had completed V6.

### 2.7. Adverse Event Reporting and Safety Monitoring

Adverse events (AE) were reported by the investigators and coded for medical diagnosis, severity, and likely causality/relatedness to the study formulas. AE were independently verified by the medical monitor before unblinding of the study. Based on groups of related diagnostic terms defined in the Medical Dictionary for Regulatory Activities (MedDRA), ‘AE of interest’ were defined for lower respiratory tract infection (LRTI), upper respiratory tract infection (URTI), otitis media, gastrointestinal infection/acute diarrhea, urinary tract infection, and other viral infections. 

### 2.8. Statistical Analysis

The intent-to-treat (ITT) analysis set comprised all infants who were randomized. The full analysis set (FAS) included all randomized infants who also commenced the allocated treatment formula. The per protocol (PP) analysis was limited to those who completed the study from enrolment to V4 (primary endpoint) without major protocol deviations. All statistical analyses were performed for both the FAS and PP cohorts. The age-adjusted mean weight gain per day was calculated for each infant for the period from V0 to V4. Weight gain for both cohorts was compared by unpaired t-test for noninferiority (primary study endpoint). Weight, length, and head circumference (HC) measurements were converted into weight-for-age (WAZ), length-for-age (LAZ), HC-for-age (HCAZ) and body mass index-for-age (BMIAZ) Z scores, according to the WHO growth standards [36]. Anthropometric parameters and CoMiSS scores were compared by paired t-test between groups for each timepoint from V0 to V6. The CoMiSS change from V0 to V1 in each group was assessed by the nonparametric Wilcoxon signed rank test for paired data.Based on the adverse events reporting (AE of interest) the incidence of infections was calculated as the proportion of infant with at least one infection during the study period to V6. Incidence figures were compared by χ^2^ test between infants receiving the test or control formulas. The frequency of respiratory tract infections was calculated as mean episodes per month of study formula exposure from enrollment to 12 months of age. The mean rate of infections per month was compared between groups by two-sample t-test. Antibiotic and antipyretic use was calculated as the proportion of infants requiring at least one treatment during the study to 12 months and compared by χ^2^ test. The relative risk reduction for the incidence of specific infections was calculated for the difference between the test and control groups. 

### 2.9. Sample Size Calculation 

The sample size to demonstrate noninferiority of the test formula for weight gain per day was based on the standard deviation of σ = ±6.1 g/day observed in a growth study by Puccio et al. [22]. A one-sided test with a noninferiority margin of Δ = −3 g/day (at an α-level of 0.025 and with 90% power) required a sample size of at least 65 infants in each group. Allowing for 25% noncompleters, we aimed to enroll at least 194 infants. 

## 3. Results

Of 200 infants screened, 194 (Poland, *n* = 100; Italy, *n* = 32; United Kingdom, *n* = 30; Spain, *n* = 12; Hungary, *n* = 10; Belgium, *n* = 7; and Singapore, *n* = 3) were randomized to receive either the test (*n* = 97) or control formula (*n* = 97). Of these, 94 infants in the test and 96 in the control group commenced taking the allocated formula (FAS cohort). None of the infants underwent skin prick testing or measurement cow’s milk-specific serum IgE prior to enrollment. Demographics and clinical details of infants at the time of enrollment are summarized in Table 1. 

**Figure 1:**
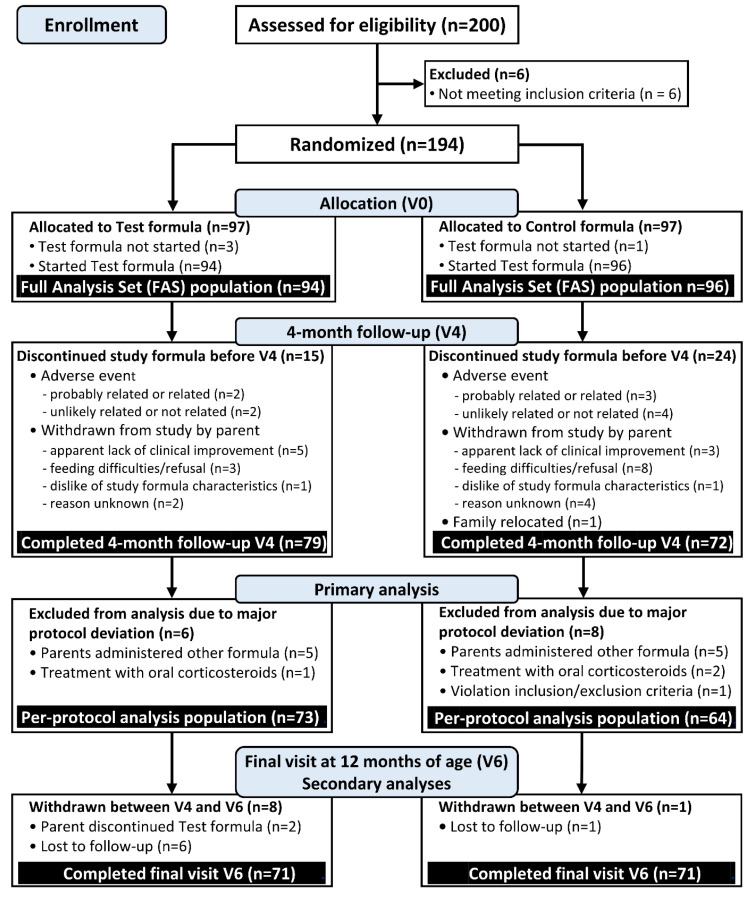
CONSORT study flow diagram.

At V4, 73 infants assigned to the test and 64 assigned to control formula completed the protocol without major deviations (PP cohort). The recruitment process and study flow of participants is summarized in the CONSORT study flow diagram; Figure 1.

### 3.1. Weight Gain per Day (Primary Endpoint)

For the per-protocol (PP) analysis set, the adjusted mean weight gain from baseline to V4 in the HMO was 19.4 g/day (95% confidence interval [CI], 17.31–21.45), while infants in the control group gained 20.1 g/day (95% CI, 17.96–22.29). The mean weight difference between groups was –0.74 g/day (1-sided 97.5% CI, –2.45–∞). This confirmed the noninferiority of the test formula regarding daily weight gain; *p* = 0.0049. A sensitivity analysis for the full analysis set (FAS) yielded the same conclusion: HMO group 19.58 g/day (95% CI, 17.9–21.3) vs. control group 19.1 g/day (95% CI, 17.4–20.9); mean difference 0.45 g/day (1-sided 97.5% CI, –1.22–∞); *p* < 0.0001.

### 3.2. Anthropometric Data

**Figure 2:**
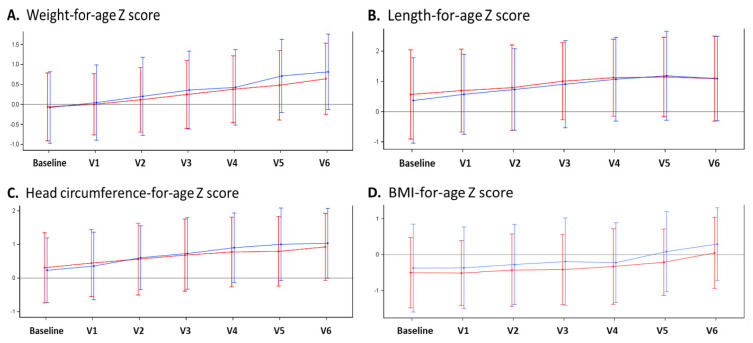
Mean Z scores with 95% confidence intervals from baseline to 12 months of age (V6). Human milk oligosaccharides (HMO) group shown in red; control group in blue. (**A**) Weight-for-age Z score; (**B**) length-for-age Z score; (**C**) head circumference-for-age Z score; and (**D**) BMI-for-age Z score.

Comparing mean anthropometric parameters between the test and control groups, no significant differences in body weight, body length, head circumference, or BMI were observed at any timepoint from V0 (baseline) to V6 (i.e., 12 months of age). In addition, there were no significant group differences for weight-for-age, length-for-age, head circumference-for-age, or BMI-for-age Z scores. In both groups, we observed an upward trend of all Z scores by between 0.5 to 1 standard deviation from enrolment to 12 months (V6), suggesting a minor increase in weight gain and growth velocity compared to that of the WHO growth reference. The anthropometric measurements from baseline to V6 are summarized in Figure 2.

### 3.3. Formula Intake

In the FAS analysis cohort, the median duration of study formula intake from enrollment to V4 was 17.3 weeks (range 0.3–19.3) in the HMO group, and 17.3 weeks (range 0.1–18.7) in the control group. From enrollment to the final visit at 12 months of age (V6), infants in the HMO group received formula for a median of 32.8 weeks (range 0.3–53.1), and control infants for 30.2 weeks (range 0.1–54.9). The PP population at V6 had the longest exposure time to study formula with a median of 35.9 weeks (range 17.3–53.1) in the HMO group, and 36.9 weeks (range 19.3–54.9) in the control group. 

In the FAS population, mean formula intake volumes for the HMO vs. control groups were similar at V1 (867 ± 207 mL/day vs. 857 ± 197 mL/day; *p* = 0.764) and V2 (904 ± 172 mL/day vs. 852 ± 237 mL/day; *p* = 0.135). Mean intakes for subsequent visits were between 10%–13% higher in the HMO group at V3 (906 ± 198 mL/day vs. 812 ± 223 mL/day; *p* = 0.009), V4 (878 ± 252 mL/day vs. 767 ± 214 mL/day; *p* = 0.006) and V5 (783 ± 345 mL/day vs. 694 ± 225 mL/day; *p* = 0.047). At V6, formula intakes were similar again between groups (631 ± 248 mL/day vs. 569 ± 225 mL/day; *p* = 0.233).

### 3.4. Resolution of CMPA Symptoms

**Figure 3:**
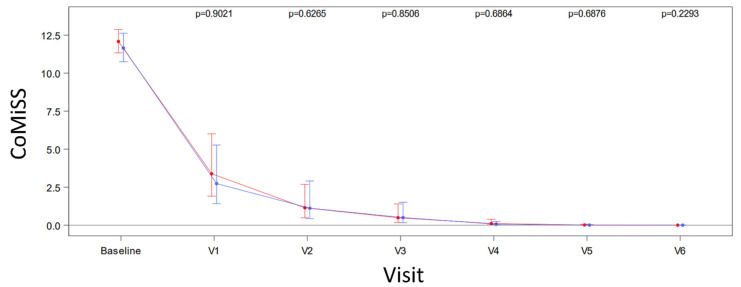
Cow’s milk-related symptom score (CoMiSS) from baseline to 12 months of age (Visit 6; V6). HMO group shown in red; control group shown in blue. Error bars indicate 95% confidence intervals.

The symptom response to treatment with either study formula was assessed by the CoMiSS tool. Overall, there were no significant group differences in CoMiSS at any of the study timepoints. In both study groups, CoMiSS decreased significantly by a median score of –6 (interquartile range [IQR] HMO group –19; +3 vs. control group –19; +4; paired Wilcoxon signed rank test *p* < 0.0001). In the HMO group, the adjusted geometric mean of CoMiSS fell from 12.08 (95% CI, 11.34–12.88) at baseline to 3.38 (95% CI, 1.91–2.69) at V1, and there was a similar decrease from 11.65 (95% CI, 10.75–12.63) to 2.73 (95% CI, 1.42–2.91) in the control group. Over the remainder of the study period from V2 to V6, there was a further progressive fall in CoMiSS in both groups, reaching a median CoMiSS of 0 at V5; Figure 3.

### 3.5. Safety

The safety analysis was based on the FAS cohort. Overall, the rate of adverse events (AE) was similar for both study groups. In the primary study period to V4, 143 AE (serious and nonserious) were reported in 60 (63.8%) infants assigned to the HMO group, and 144 AE occurred in 60 (62.5%) control infants (OR 1.06; 95% CI, 0.56–1.99; *p* = 0.88). Between V4 and V6, 37 (39.4%) infants in the HMOs group experienced 79 AE, and 97 AE were reported in 36 (37.5%) control infants (OR 1.08; 95% CI, 0.58–2.02; *p* = 0.88).

From enrollment (V0) to 12 months of age (V6), there were 14 and 12 serious AE (SAE) in the HMO and control groups, respectively. In the HMO group, these included seven infants with acute infections (gastroenteritis/diarrhea, *n* = 4; urinary tract infection, *n*=1; laryngitis/croup, *n* = 1; bronchiolitis, *n* = 1), four infants with functional problems (poor feeding, *n* = 2; persistent crying, *n* = 1; poor weight gain, *n* = 1), two infants with febrile convulsions, and one with apneic episodes. In the control group, there were five infants with acute infections (upper respiratory tract infection, *n* = 1; lower respiratory tract infection/bronchiolitis, *n* = 2; gastroenteritis, *n* = 1; viral infection, *n* = 1), three infants with respiratory problems (apneic episodes, *n* = 1; bronchial obstruction, *n* = 1; choking, *n* = 1), one infant with cyanotic heart disease, one with seizure/syncope, one with vomiting (cause not specified), and one infant admitted to hospital for a diagnostic procedure.

Based on the investigator assessment of the relatedness of any AE to study product, there was one AE (1.1%) in each study group that was deemed ‘related’ (HMO group: vomiting, *n* = 1; control group: increased regurgitation, *n* = 1). In addition, in the HMO group investigators rated as 5 AE in 4 (4.3%) infants (constipation, *n* = 2; diarrhea, *n* = 1; eczema, *n* = 1) as ‘probably related’, compared to 10 AE in 6 (6.3%) infants (persistent crying with increased regurgitation, *n* = 2; increased regurgitation, *n* = 1; vomiting, *n* = 1; vomiting and diarrhea with perianal excoriation, *n* = 1; constipation, *n* = 1) in the control group. None of the AE reported between V4 and V6 were reported as ‘related’ or ‘probably related’. Furthermore, none of the SAE were deemed ‘related’ to the study formulas, and there were no reports of anaphylaxis.

### 3.6. Morbidity

**Figure 4:**
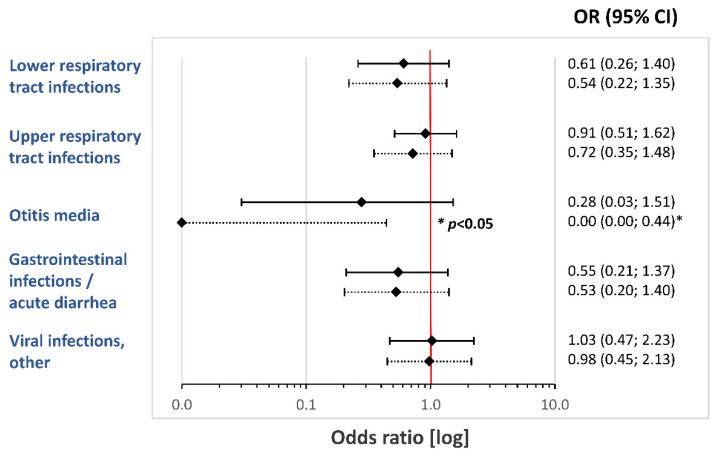
Forest plot summarizing odds ratios (95% confidence intervals; CI) for risk of developing at least 1 infection from enrollment (V0) to 12 months of age (V6). Solid lines refer to FAS, and dotted lines to per protocol (PP) analysis cohort.

Infants receiving HMO-supplemented formula experienced a numerically lower rate in respiratory and gastrointestinal infections, compared to that of infants in the control group. These group differences for the FAS cohort did not reach statistical significance. The odds ratios for the risk of various infections from enrollment (V0) to the age of 12 months (V6) are summarized in Figure 4.

#### 3.6.1. Lower Respiratory Tract Infections (LRTI)

Based on the FAS, 13 of 94 (13.8%) infants in the HMO group and 20 of 96 (20.8%) control infants developed at least one LRTI episode from enrollment to V6. This represents a relative risk reduction of 33.7% (OR 0.61; 95% CI, 0.26; 1.40; *p* = 0.251).

#### 3.6.2. Upper Respiratory Tract Infections (URTI)

In the FAS cohort, the HMO group recorded 60 URTI episodes in 39 (41.5%) infants, compared to 94 episodes in 42 (43.8%) infants (OR 0.91; 95% CI, 0.49–1.69; *p* = 0.771). Due to the similar URTI incidence in both groups, there was only a minor relative risk reduction of 5.2% for the HMO group. However, we found a significant difference in the frequency of URTI episodes. In the HMO group, infants had on average 1.5 episodes in the first year of life, compared to 2.2 episodes in the control group. When performing a *post hoc* analysis on the frequency of URTI episodes per month of study formula exposure from V0 to V6, we found a significant reduction in the frequency of URTI episodes in the HMO group (HMO 0.09 [95% CI, 0.07–0.11] vs. control 0.15 [95% CI, 0.12–0.19] episodes per month; hazard ratio (HR) 0.58 [95% CI, 0.41–0.83]; *p* = 0.003).

#### 3.6.3. Otitis Media

A total of nine ear infections/otitis media were reported from enrollment to V6 (HMO 2 of 94 [2.1%] vs. control 7 of 96 [7.3%]; OR 0.28; 95% CI, 0.03–1.51; *p* = 0.17). This corresponds with a 70% relative risk reduction in otitis media in the first year of life. On exploratory analysis of infants who completed the study to V4 (PP cohort), there were 0 infants with ear infections in the HMO group, compared to 4 (6.3%) in the control group (OR 0.00; 95% CI, 0.00–0.95; *p* = 0.045).

#### 3.6.4. Gastrointestinal Infections/Acute Diarrhea

In the HMO group, 10 (10.6%) infants developed episodes of acute diarrhea/gastrointestinal infection, compared to 17 (17.7%) in the control group (OR 0.55; 95% CI, 0.21–1.37; *p* = 0.213). This corresponds with a relative risk reduction of 40.1%.

#### 3.6.5. Other Viral Infections

Numbers of other, nonspecified viral infections with fever or rashes were similar between treatment groups (HMO 19 [20.2%] vs. control 19 [19.8%] infants; OR 1.03; 95% CI, 0.47–2.23; *p* = 1.00).

#### 3.6.6. Urinary Tract Infections

Between V0 and V6, 4 (4.3%) subjects in the HMO group were diagnosed with a urinary tract infection, compared to none in the control group (*p* = 0.058).

#### 3.6.7. Antibiotic and Antipyretic Use

Overall, antibiotic use in the FAS population from enrollment to V6 was similar between both study groups, with 23 (24.5%) infants in the HMO group and 25 (26.0%) infants in the control group receiving systemic antibiotics (OR 0.92; 95% CI, 0.47–1.81; *p* = 0.819). Only 15 (16.0%) and 14 (14.6%) infants required antibiotics before V4 in the HMO and control groups, respectively (OR 1.16; 95% CI, 0.52–2.60; *p* = 0.723). 

There was no significant difference in antipyretic use from V0 to V6, with 35 (37.2%) infants in the HMO group, and 40 (41.7%) infants in the control group receiving antipyretic medications (OR 0.85; 95% CI, 0.46–1.57; *p* = 0.605). Prior to V4, antipyretic use was similar between groups (HMO group 28 [29.8%] vs. control group 31 [32.3%]; OR 0.95; 95% CI, 0.49–1.84; *p* = 0.890). By contrast, infants in the HMO group required significantly fewer antipyretics between V4 and V6 (HMO group 18 [22.8%] vs. control group 26 [36.1%]; OR 0.38; 95% CI, 0.17–0.84; *p* = 0.017).

## 4. Discussion

The present randomized trial confirmed that the test formula with two HMO and reduced protein content supports normal growth in infants with CMPA. The formula was tolerated well, and the safety profiles of the test and control formulas were similar. In addition, there was a highly significant reduction in CMPA symptoms, as evidenced by a fall in CoMiSS to levels reported in healthy infants [32]. Noninferiority of the test formula for weight gain per day was confirmed at the 4-month visit. There were no significant group differences for any of anthropometric parameters (weight, length, head circumference, BMI) at any of the study visits. We observed a minor upward trend in growth parameters from enrollment to 12 months of age, compared to that of the WHO growth reference [36]. Accelerated growth compared to that of breastfed infants was observed in multiple studies in formula-fed infants [27]. In the early stages of the present study, this possibly indicated catch-up growth and nutritional recovery from CMPA after commencing an EHF. However, in the second half of the first year, growth parameters are not only related to the study formula but significantly influenced by the complementary diet. As the current study did not collect detailed information on the type and amount of the complementary feeding, it was not possible to assess the relative contribution of formula vs. complementary diet on the accelerated growth.

The test group received less protein from the study formula in an attempt to move the protein intake closer to that of breastfed infants [25]. This is thought to have a beneficial effect on the long-term risk of obesity via early metabolic programming [37]. Mechanistic studies in infants suggested that this is modulated via an increase in serum acylcarnitines, which may stimulate beta-oxidation and fat storage [26]. However, data from a randomized trial between two formulas with standard and low protein found no statistical differences for growth parameters at 4 years of age [38]. Importantly, in the present study we did not observe any significant group differences in anthropometric parameters at 12 months of age. The addition of HMO is unlikely to have a direct effect on weight gain and growth, as only 1%–2% of HMO is absorbed. Indirect short- and long-term beneficial effects of HMO on growth parameters were proposed, which require further evaluation in longitudinal trials [39,40].

The addition of 2′-FL and LNnT was associated with a reduction in a range of infections. Beneficial effects of HMO include the reduction in glycan-dependent viral infections (e.g., rotavirus), as well as an increased production of short-chain fatty acids (SCFA) by bifidobacteria, which create an acidic gut environment and protect from invasive enteropathogens [3,14,41]. The main HMO species in the breast milk of secretor mothers is 2′-FL, and it persists at levels of around 1g/L until about 12 months of age [18]. The test formula contained similar amounts of 2′-FL, and an anti-infective effect comparable to that seen in breastfed infants thus appears plausible. In addition, experimental data on LNnT and its derivatives suggest a protective effect against pneumococcal pneumonia by decreasing pneumococcal load in the lungs and reducing the risk of bacteremia [42]. As an unexpected finding, we report four infants with urinary tract infections in the HMO group, compared to none in the control group. However, it appears unlikely that this is related to the test formula or added HMO. In a previous mechanistic study, HMO appeared to protect against bladder infection with uropathogenic *Escherichia coli* [43]. Our study is the first to demonstrate a reduced frequency of URTI during the first year of life. Ear infections were also less common in the HMO group. In addition, there was a nonsignificant reduction in LRTI and episodes of acute diarrhea in the HMO-supplemented group. Our findings are in line with data from an earlier study on healthy infants receiving a cow’s milk-based formula supplemented with 2′-FL and LNnT, which found significantly fewer LRTI and a reduced need for antibiotics in the first year of life [22].

The present study had several limitations. The diagnosis of CMPA was based on a combination of clinical criteria but not confirmed by a diagnostic oral food challenge (OFC) [44]. However, the high baseline CoMiSS of around 12 and significant fall by 6 points after the elimination diet makes a diagnosis of CMPA probable. A study assessing the diagnostic accuracy of CoMiSS found that a score of 12 or above was highly specific and predictive of CMPA [45]. It therefore appears likely that most infants in this study suffered from CMPA. Another limitation relates to the ascertainment of infection rates during the study period. The analysis was based on the AE reporting for the FAS cohort by clinicians. AE were recorded retrospectively at each study visit. This possibly introduced recall bias, particularly for V5 and V6, which in some infants occurred several months apart. Overall, the number of reported infections appeared quite low. By comparison, a recent German clinical trial reported much higher incidence figures for LRTI, URTI, and otitis media in the first year of life (29.6%, 66.2%, and 89.8%, respectively) [46]. This may indicate a degree of underreporting in the present study due to the mechanism by which infections were captured. While a numerically large relative risk reduction of 30%–40% was found for LRTI and gastrointestinal infections, the low overall infection incidence and limited sample size was insufficient to reliably assess these secondary outcomes.

## 5. Conclusions

The present study confirmed that the test formula with human milk oligosaccharides (HMO) and reduced protein content supports normal growth in infants with cow’s milk protein allergy (CMPA). Both study formulas effectively controlled CMPA symptoms within 1 month (V1). The test formula was tolerated well, with a safety profile comparable to that of the existing extensively hydrolyzed formula (EHF) without HMO. Infants receiving the HMO-supplemented EHF appeared to have a significantly lower frequency of upper respiratory tract infections (URTI), as well as a lower risk of otitis media. In addition, the HMO-containing formula was associated with a nonsignificant reduction in lower respiratory tract infections, gastrointestinal infections, as well as medication use. However, sample size limitations did not allow firm statistical conclusions on these secondary outcomes. Further studies are needed to confirm these findings and explore the mechanisms by which HMO reduce the risk of respiratory and gastrointestinal infections in infancy.

## Figures and Tables

**Table 1:** Baseline characteristics of study participants who commenced treatment (full analysis set, FAS).

Infant Characteristics	Test Formula Group (*n* = 94)	Control Formula Group (*n* = 96)
Age, months ^1^	3.2 ± 1.7	3.2 ± 1.7
Gestational age, weeks ^1^	38.9 ± 1.1	39.2 ± 1.2
Male sex, *n* (%)	48 (51.1)	48 (50.0)
Birth by Cesarean section, *n* (%)	46 (48.9)	45 (46.9)
Race		
Caucasian, *n* (%)	93 (98.9)	93 (96.9)
Asian, *n* (%)	1 (1.1)	3 (3.1)
Anthropometric measurements ^1^		
Weight, kg	5.95 ± 1.37	5.95 ± 1.34
Length, cm	61.5 ± 5.66	61.1 ± 5.25
Head circumference, cm	40.1 ± 2.55	40.3 ± 2.36
Symptoms at enrolment		
Persistent crying, *n* (%)	82 (87.2)	81 (84.4)
Regurgitation, *n* (%)	82 (87.2)	79 (82.3)
Abnormal stools (liquid stools or constipation, *n* (%)	79 (84.0)	84 (87.5)
Atopic dermatitis, *n* (%)	68 (72.3)	64 (66.7)
Urticaria, *n* (%)	7 (7.4)	5 (5.2)
Respiratory symptoms, *n* (%)	44 (46.8)	48 (50.0)

^1^ Mean ± standard deviation.

## Data Availability

Data are available on request from the Chief Science & Medical Officer, Nestlé Health Science, 1800 Vevey, Switzerland.
